# Balancing Privacy and Utility in Child and Adolescent Mental Health Services Research: Retrospective Cohort Study on Synthetic Data Generation

**DOI:** 10.2196/71819

**Published:** 2026-02-26

**Authors:** Mounir Haizoune, Bennett L Leventhal, Dipendra Pant, Øystein Nytrø, Kaban Koochakpour, Roman A Koposov, Lars Ravn Øhlckers, Norbert Skokauskas

**Affiliations:** 1 Department of Analytics Western Norway Regional Health Authority Stavanger Norway; 2 Department of Psychiatry and Behavioral Neuroscience University of Chicago Chicago, IL United States; 3 Norwegian University of Science and Technology Trondheim Norway; 4 Department of Child and Adolescent Psychiatry, Clinic of Mental Health Care St. Olav University Hospital Trondheim Trondheim Norway; 5 Regional Centre for Child and Youth Mental Health and Child Welfare, Faculty of Health Sciences UiT The Arctic University of Norway Tromsø Norway; 6 Department of Child and Adolescent Psychiatry and Addiction Stavanger University Hospital Stavanger Norway; 7 Regional Center for Child and Youth Mental Health and Child Welfare Faculty of Medicine and Health Science Trondheim Norway

**Keywords:** CAMHS, classification, length of referral, mental health, synthetic data generation

## Abstract

**Background:**

Electronic health records are essential for advancing research aimed at improving clinical outcomes. However, stringent data protection and privacy concerns severely limit the accessibility and use of real clinical data, particularly within Child and Adolescent Mental Health Services (CAMHS) involving vulnerable young individuals. This challenge can be effectively addressed through synthetic data generation, which safeguards individual privacy while facilitating comprehensive analyses of clinical information.

**Objective:**

This study aims to investigate whether hierarchical synthetic data generators (SDGs) can effectively replicate the statistical properties, preserve the utility, and maintain the privacy of real CAMHS clinical data, thereby enabling data sharing and broader access to research-ready datasets.

**Methods:**

This retrospective cohort study used electronic medical record data from 6924 distinct patients from CAMHS in Stavanger, Norway, comprising 7730 referral periods and 58,524 episodes of care. An 80%-20% split was used for training and testing. A hierarchical synthetic data generation model was trained to generate synthetic referral periods and associated episodes of care. Data quality was evaluated using SDMetrics for distribution (Kolmogorov-Smirnov Complement [KSC]/Total Variation Complement [TVC]), correlation (CorrelationSimilarity [CS]), and cardinality (CardinalityShapeSimilarity [CSS]) similarity. Privacy was evaluated using the Anonymeter library to simulate singling out, linkability, and inference reidentification attacks. Utility was assessed using the train synthetic test real (TSTR) pattern, comparing the predictive performance using precision-recall area under the curve [PRAUC] of models trained on synthetic vs real data for classifying the intensity of care.

**Results:**

The hierarchical SDG created highly reproducible synthetic CAMHS data. The average statistical similarity scores were high across all metrics: KSC/TVC at 0.92, CS at 0.77 (intertable CS at 0.75), and CSS at 0.92. The synthetic data also demonstrated a low risk under simulated privacy attacks on a control dataset (n=1546): the average success rate was 6/1546 (0.39%) for singling out and 77/1546 (5%) for multivariate attacks. The average linkability risk was 54/1546 (0.5%), and the highest inference risk for a sensitive variable was 2/1546 (0.12%). The classification model trained on synthetic data (TSTR) produced comparable predictive performance (PRAUC=0.40) to the model trained on real data (PRAUC=0.43) for classifying the intensity of care (low vs medium or higher). Shapley additive explanations analysis confirmed that the synthetic model’s explanations aligned with real-world insights, validating its ability to capture fundamental predictive patterns.

**Conclusions:**

Synthetic data can be used to build trust and promote collaboration among CAMHS researchers by offering access to extensive, representative samples with a low risk of patient identification. This approach expands the breadth of research while safeguarding patient privacy. Effective implementation of synthetic data generation depends on the model’s ability to accurately identify and replicate the complex, sequential patterns present in real data.

## Introduction

The preservation of patient confidentiality is frequently identified as a significant barrier to conducting research with mental health care datasets [1]. Researchers appropriately face strict regulations and ethical considerations that limit access to real patient data. This is further complicated by selection bias [2], digital barriers [3], and high levels of sensitivity of mental health information, which, if disclosed, could lead to additional stigma, discrimination, and other negative consequences for individuals [4].

While these challenges are present across all mental health research, the use of large-scale data from patient records becomes significantly more complex with Child and Adolescent Mental Health Services (CAMHS) data. This is due to a set of problems associated with this vulnerable population. First, the variability in developmental stages among children and adolescents means that symptoms and diagnoses manifest differently across age groups (eg, anxiety in a 5-year-old compared to a 16-year-old), making data standardization and longitudinal analysis inherently difficult. Second, the necessity for age-appropriate interventions and evolving care pathways creates complex, nonlinear data structures that are challenging to model consistently [5]. Perhaps most critically, the ethical considerations surrounding privacy and consent for minors are far more stringent than for adult populations. Researchers must navigate nuanced consent processes involving both parents or guardians and the child’s assent, often with varying legal requirements based on age and local jurisdiction [6,7]. This multilayered consent, coupled with heightened safeguarding responsibilities, severely restricts data sharing. Furthermore, due to their inherent nature, which includes stringent privacy regulations and the fragmented structure of care pathways, CAMHS datasets are typically smaller, exhibit greater structural fragmentation, and are consequently more challenging to access large-scale, generalizable research than adult health datasets.

These challenges can be effectively addressed by the generation of synthetic data. Synthetic data not only safeguard individual privacy but also permit comprehensive analyses of large-scale clinical data obtained directly from electronic medical records (EMRs) and other clinical data sources, even in sensitive areas such as CAMHS.

Previous studies have applied synthetic mental health data in data augmentation, privacy preservation, and fairness [8]. The SynthNotes framework, developed by Begoli et al [9], is notable for its ability to produce realistic and high-fidelity synthetic mental health documentation on a large scale. The framework facilitates benchmarking, optimization, and training for biomedical NLP, information extraction, and machine learning systems, all while ensuring strict privacy preservation. To ensure accurate and generalizable research results, synthetic data must accurately represent diverse minority subgroups. This is particularly important in addressing child and adolescent mental health inequities exacerbated by events like the COVID-19 pandemic [10]. Addressing these biases requires expertise in the underlying model and an understanding of fairness concepts like unawareness, demographic parity, and conditional fairness [8]. One approach involves directly de-biasing existing datasets to create “fair data,” a process similar to synthetic data generation [11,12]. Alternatively, some researchers view data de-biasing as a “ground-up” generation problem, focusing on developing generative models that inherently incorporate fairness [13].

Predictive models trained on synthetic data for mental health have yielded encouraging findings in numerous studies, highlighting the critical importance of data quality, ethical considerations, and the necessity for ongoing research. A study by Rankin et al [14] used a deep learning approach to predict mental health quality using synthetic data derived from functional network connectivity data from resting-state functional magnetic resonance imaging, achieving high accuracy in classifying mental health quality. Using models trained on synthetic data, Ajith et al [15] combined data from multiple longitudinal mobile sensing studies to predict mental health symptoms, showing improved generalization across different datasets. While these models frequently failed to capture intricate patterns and subtleties, especially within diverse populations [16], their results highlight the potential of synthetic data to enhance predictive modeling in mental health, while addressing privacy and fairness concerns.

While prior synthetic health data studies focused on adult mental health, our research specifically examines how synthetic data generators (SDGs) effectively capture complex patterns and relationships in CAMHS data. We assess the essential trade-offs involving data quality, patient privacy, analytical utility, and optimal synthetic sample size to ensure generated data remains representative and valuable for research.

## Methods

### Data Source

This study was conducted using a retrospectively collected cohort from the Child and Adolescent Mental Health Department (CAMHD) at Stavanger University Hospital (SUS). The hospital functions as a referral center for about half a million inhabitants in the Rogaland metropolitan area and had an annual admission ranging from 17,000 to 19,000 during the study period. CAMHD at SUS provides assessment, diagnosis, and treatment for young people aged 18 years or younger, experiencing mental health issues such as depression, anxiety, trauma, developmental disorders like autism or attention-deficit/hyperactivity disorder (ADHD), and psychosis.

As detailed in Table 1, the dataset included 6924 distinct patients, 7730 referral periods, and a total of 58,524 episodes of care. Data extraction was performed using the electronic health record system (Distrubuert Informasjons og Pasientdatasystem i Sykehuset [DIPS]; Helse Vest IKT). The system provided access to administrative details like time of admission, department, and clinical personnel, as well as clinical information such as age, sex, and diagnosis for each episode of care.

Each episode is directly linked to a specific referral through a unique identifier. Allowing for comprehensive and continuous tracking of a patient’s journey from their initial referral through all subsequent clinical interactions.

**Table 1:** Overview of the Child and Adolescent Mental Health Services dataset.

Variable	Entity	Description	Descriptive statistics
referralID	Consultation/stay	Unique identifier for each referral	N/A^a^
Patientage	Referral	Age of the patient at referral	Mean 11 (SD 4) Median 12 (IQR 15)
Patientsex	Referral	Patient’s sex (0=female; 1=male)	Unique values=2 Most frequent=male
referralStartDate	Referral	Date the referral was initiated	Minimum=2014 Maximum=2018
referralLength	Referral	The duration of the referral period in days	Mean 58 (SD 41) Median 30 (IQR 60)
stayID	Consultation/stay	Unique identifier for each consultation or stay	N/A
staySartDate	Consultation/stay	Date when the consultation or stay began	Minimum=2014 Maximum=2018
StayLength	Consultation/stay	Duration of each stay in minutes	Mean 58 (SD 41) Median 30 (IQR 60)
personnelGrp	Consultation/stay	Category of medical personnel involved	Unique values=4 Most frequent=“psychologist”
stayTypeGrp	Consultation/stay	Grouped type of consultation or stay	Unique values=5 Most frequent=“treatment”
specialist	Consultation/stay	Indicates if the medical personnel is a specialist (yes/no)	Unique values=2 Most frequent=“no”
roleGrp	Consultation/stay	Role of the clinical personnel during the consultation or stay	Unique values=2 Most frequent=“contact”
cond1axisIGrp	Consultation/stay	*ICD-10*^b^ Axis I (clinical syndromes)	Unique values=8 Most frequent=“R40-R46”
cond1axisIIGrp	Consultation/stay	*ICD-10* Axis II (disabilities)	Unique values=4 Most frequent=N/A
cond1axisIIIGrp	Consultation/stay	*ICD-10* Axis III (intellectual developmental disorder)	Unique values=4 Most frequent=N/A
cond1axisIVGrp	Consultation/stay	*ICD-10* Axis IV (somatic diseases)	Unique values=4 Most frequent=N/A
cond1axisVGrp	Consultation/stay	*ICD-10* Axis V (psychiatric disorders and somatic diseases)	Unique values=6 Most frequent=N/A
cond1axisVIGrp	Consultation/stay	*ICD-10* Axis VI, global functioning (CGAS^c^)	Unique values=7 Most frequent=6999

^a^N/A: not applicable.

^b^ICD-10: International Statistical Classification of Diseases, Tenth Revision.

^c^CGAS: Children Global Assessment Score.

### Missing Data

The dataset contains *ICD-10* (*International Statistical Classification of Diseases, Tenth Revision*) diagnostic codes categorized across different axes or levels of detail. The *ICD-10* uses a multiaxial system for mental and behavioral disorders, including Axis I (clinical syndromes), Axis II (disabilities), and Axis III (environmental/lifestyle factors).

A key characteristic of the Axis columns is the high rate of missing data, which indicates “not applicable” rather than an error, as is common in clinical coding. For instance, the first condition in Axis I is nearly complete (0.7% missing), but subsequent codes within the same axis show drastically increased missingness (eg, 6th condition, 99.8% missing). This trend continues across other axes, like Axis II (eg, 3rd condition 99.6% missing). This consistent pattern of missing values signifies that a code was not relevant to a clinical episode, making data imputation inappropriate.

### Ethical Considerations

The study is a quality assurance initiative directly building on the results of the “PILOT SYNDATA” project. The project was conducted as a collaboration between CAMHD SUS, the analytical department at SUS, and Helse Vest IKT (the regional IT service delivery company). The project’s main purpose was to explore the safe use of synthetic data in mental health care.

The data for the “PILOT SYNDATA” project were collected from DIPS, and Helse Vest IKT performed the deidentification and aggregation procedures, with the resulting anonymized data then being used for synthetic data generation in the next phase. This entire process was conducted under a formal agreement that permits the secondary use of health data for research and quality assurance while ensuring patient privacy and confidentiality. Due to the complete deidentification and aggregation of the data, a waiver was not required to use the data in this study.

The study used a synthetic data generation process that creates entirely new, statistically representative data points. The process is fully compliant with HIPAA (Health Insurance Portability and Accountability Act) and GDPR (General Data Protection Regulation) regulations, significantly reducing the risk of an attacker reidentifying individuals or uncovering sensitive patient details from the original dataset.

As this was a retrospective analysis of existing data, no financial compensation was provided to participants. Additionally, it is not possible to identify any individual participants in the figures or supplementary materials. The images are illustrative and use a “real-like path” to represent an anonymized, generalized patient journey.

### Synthetic Data Generation

We use HMASynthetiser, a multitable algorithm within the Synthetic Data Vault framework [17]. HMASynthetiser is a class model that implements a “hierarchical modeling algorithm” to analyze data structured in multiple levels, often called nested data. These models are particularly useful when dealing with complex datasets in which observations are grouped at different levels, such as referral periods and episodes of care (Multimedia Appendix 1).

The SDG systematically processes the real CAMHS dataset, examining how information is structured across the referrals and episodes of care tables. It uses a Gaussian copula to map the complex relationships between every data field. A Gaussian Copula [18] is a statistical model used to identify the dependency structure between the nested dataset (referrals/episodes). The approach ensures that the SDG process accurately mirrors the statistical patterns and distributions present in the original patient records, even for variables with nonstandard or highly dependent relationships. In addition, the parameters of the SDG are customized for each individual table in the hierarchy, allowing fine-tuning of the synthetic data generation process. Hierarchical modeling, combined with the statistical method, is essential for accurately capturing the complex dependency structures within CAMHS variables.

### Data Modeling

To optimize data utility and patient privacy, the real CAMHS dataset underwent comprehensive preprocessing prior to synthetic data generation. This involved the systematic categorization of variables, such as age and admission type, into broader, analytically meaningful groupings. Specifically, age was discretized into decennial intervals, while variables, such as “performing personnel” and “admission type,” were recorded by mapping their original granular values to more generalized, descriptive categories. Additionally, diagnostic Axes I-IV were grouped based on the *ICD-10* hierarchy, and Axis VI (Children's Global Assessment Score) underwent specific binning and categorization to abstract sensitive medical codes.

These comprehensive preprocessing steps ensured the data were optimally structured for generating high-fidelity synthetic data and accurately reflected real-world patterns while rigorously upholding patient data protection.

### Experimental Setup

**Figure 1:**
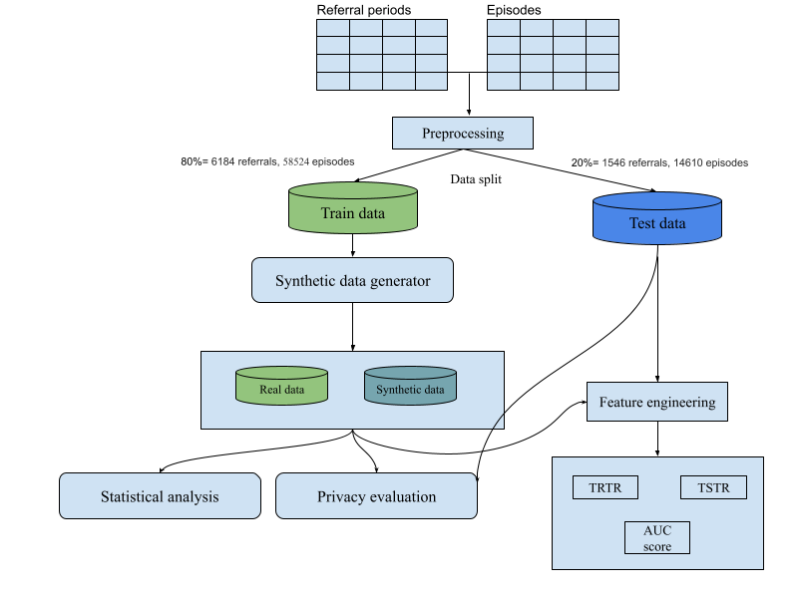
General study design. AUC: area under the curve; TRTR: train real test real; TSTR: train synthetic test real.

Figure 1 displays the experimental setup. The experiments performed in this study followed 3 structures: sample size, privacy risk assessment, and utility assessment.

#### Sample Size

To evaluate the impact of sample size on predictive model performance (Multimedia Appendix 2), 4 distinct training sets (619, 1546, 3092, and 6184 referrals) were created from the real-world CAMHS data. For each of these sizes, a corresponding synthetic dataset was generated to enable a comparison of predictive model performance using both real and synthetic data across various data availability scenarios.

#### Privacy Risk Assessment

To measure the privacy risk in the generated synthetic data, we used the experimental approach via the Python library Anonymeter [19] (Multimedia Appendix 3). Initially, the attack uses synthetic data to create a series of guesses. The model then evaluates these guesses by comparing them to the values present in the original dataset, thereby assessing the effectiveness of the attack. The privacy model requires the adversary to conduct the same attack on a control dataset (records that were not used in the SDG). By contrasting the success rates of the attack on both training records and control records, a sensitive and significant measure of risk is established. Three types of privacy risk attacks were simulated and analyzed to evaluate the potential for reidentification: singling out, linkability, and inference risks.

#### Utility Assessment

Prediction tasks represent a robust and frequently used methodology for evaluating synthetic data utility, offering distinct advantages beyond conventional statistical comparisons. The method directly confirms the data’s real-world applicability by assessing its capacity to replicate complex, nonlinear relationships for applications such as individualized mental health care planning or treatment pathway optimization. Furthermore, the approach validates the functional equivalence of synthetic data and provides a user-centric, interpretable metric of its inherent value, thereby facilitating the detection of subtle discrepancies overlooked by simpler analytical techniques [20]. To assess synthetic data utility, we formulated a downstream prediction task (Multimedia Appendix 4): classifying patient referral periods by intensity of care. Following Beckers et al [21], “intensity of care” was initially defined as low (<15 sessions/year), medium (15-51 sessions/year), or high (≥52 sessions/year). We extracted primary diagnosis of the first episode, sex, age, and total past referrals as predictor variables. Due to significant skewness (386/7730 high, 2087/7730 medium, and 5874/7730 low), the “medium intensity” and “high intensity” categories were merged into a “medium- or higher-intensity care” group. The combined group was selected to create a sufficiently sized positive class for stable model training, given the constraints of the severely skewed original distribution.

The dataset was then stratified by intensity and split into an 80% training set (6184 referral periods and 58,527 episodes) and a 20% independent test set (1546 referral periods and 14,610 episodes; Multimedia Appendix 5). The stratification ensured balanced distribution and kept all related patient data within either the training or test set; the test set was used solely for evaluation. A random forest model [22] was then trained using synthetic data generated at various scale factors (1, 2, 5, 10). The trained models were evaluated using the Train Synthetic, Test Real evaluation method [23], which involves training a model on synthetic data and testing it on real data.

Model performances were measured using precision-recall area under the curve (PRAUC), a robust metric particularly suited for imbalanced datasets [24,25]. We specifically chose PRAUC over the traditional area under the receiver operating characteristic curve because PRAUC is less susceptible to inflation from the majority (low intensity) class, providing a more accurate and rigorous measure of performance on the positive (medium- or higher-intensity care) class [24]. Results are presented as the median of a stratified 4-fold cross-validation.

Finally, SHAP (Shapley additive explanations) values [26] were computed for both models to assess variable interactions.

### Statistical Analysis

To quantify the similarity between real and synthetic data, we used 4 key metrics from the SDMetrics Python library [23]. For numerical column distributions, we used the Kolmogorov-Smirnov Complement (KSC), while the Total Variation Complement (TVC) measured discrete column distributions. The preservation of intercolumn correlation patterns was evaluated using CorrelationSimilarity (CS), and CardinalityShapeSimilarity (CSS) was applied to assess the similarity in cardinality distributions within multitable datasets.

For privacy risk evaluation, we conducted 3 attack-based assessments focused on factual anonymization indicators: singling out determined the potential for identifying individual records; linkability assessed the risk of linking synthetic data back to the real dataset; and an inference attack was used to attempt to deduce sensitive individual information from seemingly nonsensitive data. A low inference risk means it is highly unlikely that an attacker could uncover specific, private details about individual patients from the original dataset.

### Qualitative Visualization of Care Pathways

For the qualitative assessment, the specific “real patient path” was selected randomly from the overall real dataset after the synthetic data generation process was completed to ensure the visualization was not conditioned on the selected patient’s attributes prior to generation. This patient, a 12-year-old male diagnosed with “behavioral and emotional disorders” (F90-F98), represents a highly frequent and common clinical profile in our cohort. The corresponding “synthetic path” was then extracted from the synthetic data by identifying a synthetic patient with identical starting attributes (age, sex, and primary diagnosis) to provide a valid, anonymized, and attribute-matched comparison for event sequencing and density.

## Results

### Similarity and Quality Assessment of Synthetic CAMHS Data

As detailed in Table 2, the SDG produced CAMHS data that demonstrated high statistical similarity to the “real-world” CAMHS dataset. The result was evident across all reported metrics (KSC/TVC score=0.92; CS=0.75; CS (intertable)=0.73; CSS=0.9), with good fidelity remaining consistent regardless of the amount of “real-world” training samples provided to the algorithm.

**Figure 2:**
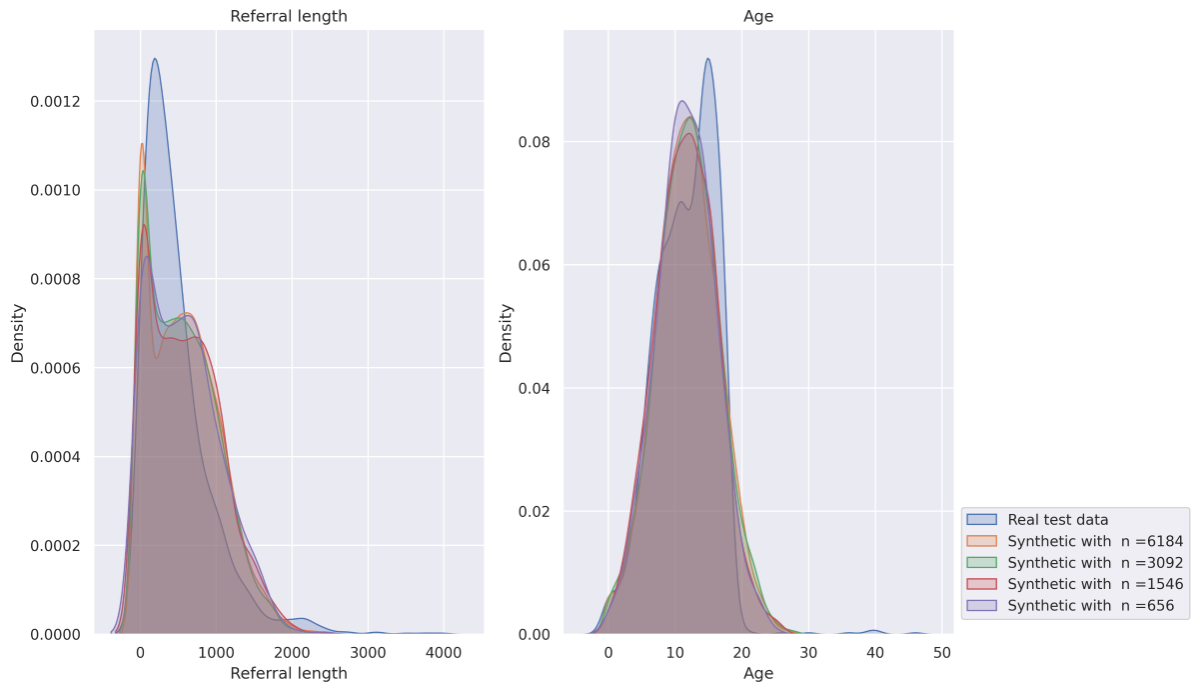
Visual comparison of the density distribution of the real and the derived synthetic Child and Adolescent Mental Health Services features. Density distributions for 2 selected features, “referral length” and “age,” and their synthetic derivatives are compared.

Figure 2 presents 2 kernel density estimate plots, offering a visual comparison of feature distributions between real-world CAMHS data and synthetic data generated at various sample sizes. The left plot, illustrating “referral length,” reveals that the synthetic data distributions, particularly those derived from larger sample sizes (n=6184 and n=3092), closely align with the real test data’s distribution in the high-density range. While the synthetic data effectively captures the predominant patterns of referral lengths, there are minor deviations observed in the extreme tails, suggesting a slight challenge in perfectly replicating infrequent, very long referrals.

Similarly, the right plot, depicting “age,” demonstrates exceptionally high fidelity. All synthetic age distributions, regardless of the sample size, exhibit remarkable overlap with the real test data’s distribution. The peaks and overall shapes are nearly identical, indicating that the synthetic data generation process is highly successful in preserving the univariate statistical characteristics of patient age.

The observed high scores across the various quality metrics and kernel density estimates provide robust evidence that the synthetic data accurately replicates the original patient information’s characteristics and inherent relationships. Specifically, the significant KSC/TVC scores confirm that individual statistical distributions of each variable within the synthetic dataset demonstrate strong consistency with those observed in the real data, which implies that features like patient age ranges or referral lengths exhibit statistical properties consistent with their real-world counterparts. Furthermore, the high CS scores, including both intratable and intertable relationships, confirm the accurate preservation of complex dependencies and interactions between distinct variables. For example, critical correlational structures, such as the frequent association of specific diagnoses with treatment pathways or demographic profiles in the real data, are accurately maintained within the synthetic dataset. Finally, the high CSS scores confirm that the structural properties of the data, including the diversity of unique values within categorical fields and the overall shape of numerical distributions, are accurately replicated.

The consistent high scores underscore that the synthetic data not only replicates individual variable characteristics but also captures the intervariable relationships and the overall statistical integrity of the original, sensitive CAMHS patient information. The observed fidelity is critical for establishing synthetic data’s suitability for subsequent analytical and research applications.

**Table 2:** Average Kolmogorov-Smirnov Complement (KSC)/Total Variation Complement (TVC), CorrelationSimilarity (CS; single and intertable), and CardinalityShapeSimilarity (CSS) metrics from synthetic data evaluation experiments using different synthetic scale factor sizes.

	656 referrals	1546 referrals	3092 referrals	6184 referrals
KSC/TVC	0.91	0.92	0.92	0.92
CS	0.73	0.77	0.75	0.77
CS (intertable)	0.69	0.73	0.73	0.75
CSS	0.87	0.90	0.91	0.92

### Synthetic Data Privacy Evaluation

To assess the potential for patient reidentification and information revelation, we performed 3 attack-based privacy evaluations: singling out, linkability, and attribute inference. Table 3 provides an overview of the success rate associated with the 3 simulated privacy attacks using synthetic data on a control real dataset (n=1546), highlighting the success rate achieved with various sample sizes. Specifically, the analysis reveals that both univariate and multivariate singling out attacks yield minimal average success rates, recorded at just 6/1546 (0.4%) and 77/1546 (5%), respectively. This indicates that the likelihood of successfully executing these types of attacks is low, regardless of the volume of data being analyzed. Furthermore, the table presents insights into the risk of linkability when using clinical and demographic variables as auxiliary data. The average risk of linkability is calculated to be 0.5% (8/1546), suggesting that while there is some potential for linking data to individuals, the overall risk remains quite low.

**Figure 3:**
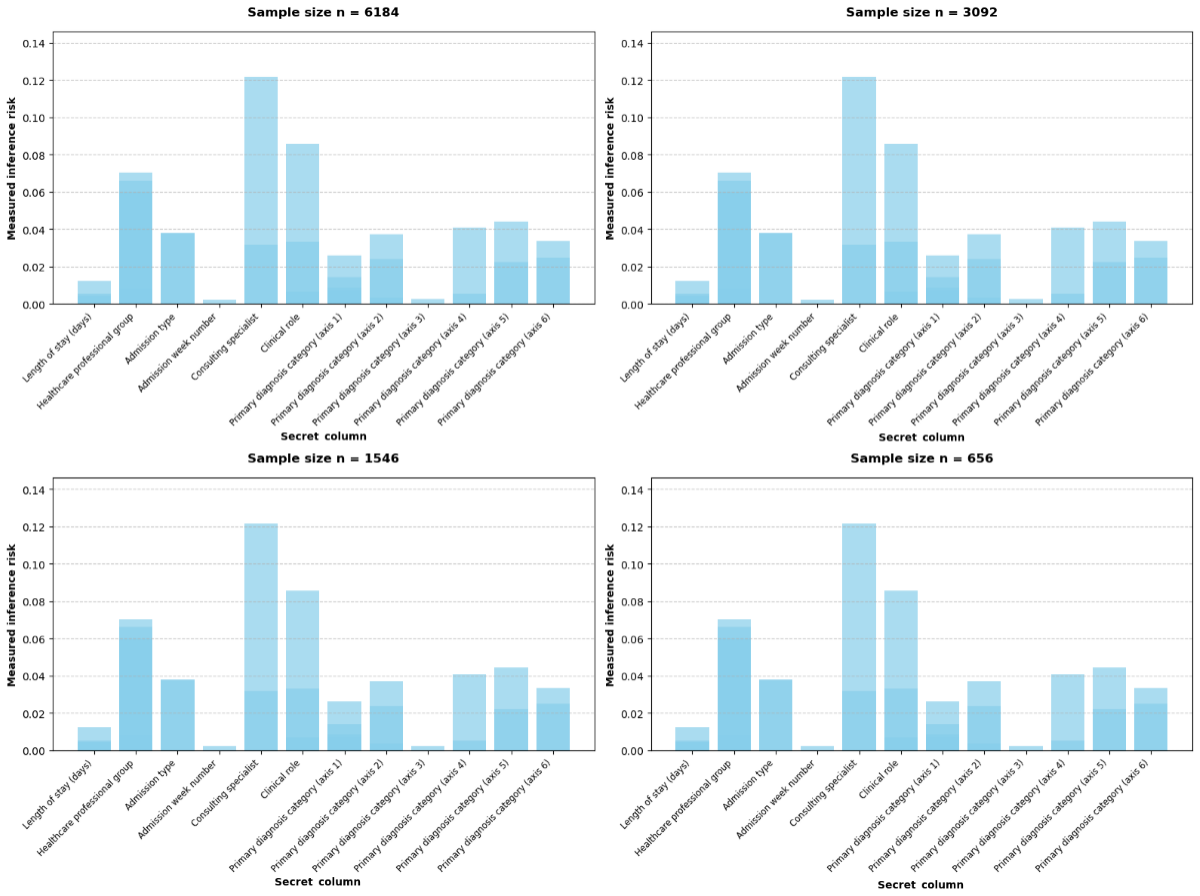
Inference risk.

Similarly, inference risk of sensitive variables from patient age and sex was minimal for most attributes (Figure 3), with “admission week number” presenting the highest, yet still low, risk (2/1546, 0.12%).

A low percentage, for instance, a range of 0%-5%, indicates that an attacker’s capacity to deduce private details from the synthetic data is only marginally superior to random chance. To contextualize this, if Figure 3 reveals an inference risk of, for example, 2%, it signifies that an attacker possessing auxiliary nonsensitive information (eg, a patient’s age and gender from an external source) would still have only a 2% likelihood of accurately determining a specific sensitive attribute, such as a rare mental health diagnosis, from the synthetic dataset. The low probability demonstrates the robust privacy preservation achieved, reinforcing the suitability of this approach for sensitive health care data.

The results clearly demonstrate the robust capability of the synthetic data to effectively protect individual privacy. The protection is provided by the synthetic data generation process, which creates entirely new, statistically representative data points rather than simply masking or perturbing original records, thereby breaking any direct link to individual patients. As a result, it becomes highly unlikely for an attacker to reidentify specific individuals or uncover private, sensitive details about patients from the original dataset, even if they possess some auxiliary information. This significantly mitigates risks associated with reidentification or attribute inference attacks, enabling a secure environment for data sharing.

**Table 3:** Success rate of singling out, linkability, and inference attacks on control data using various synthetic Child and Adolescent Mental Health Services data samples.

Risk type	6184 referrals, n/N (%)	3092 referrals, n/N (%)	1546 referrals, n/N (%)	619 referrals, n/N (%)
Singling out (univariate)^a^ success rate	6/1546 (0.38)	6/1546 (0.38)	6/1546 (0.38)	7/1546 (0.43)
Singling out (multivariate)^b^ success rate	46/1546 (3)	93/1546 (6)	62/1546 (4)	108/1546 (7)
Linkability^c^ success rate	2/1546 (0.13)	5/1546 (0.34)	11/1546 (0.69)	13/1546 (0.84)

^a^Singling out (univariate) represents the percentage of successful attempts to uniquely identify an individual in the synthetic dataset using a single characteristic (based on 500 attacks). Lower values indicate better privacy.

^b^Singling out (multivariate) represents the percentage of successful attempts to uniquely identify an individual in the synthetic dataset using multiple combined characteristics (based on 100 attacks). Lower values indicate better privacy.

^c^Linkability represents the percentage of successful attempts to link synthetic records back to their original real records (based on 2000 attacks and 10 neighbors). Lower values indicate better privacy.

### Synthetic Data Utility Evaluation

The target variable to be classified (intensity of care) in the utility evaluation was highly unbalanced in the real dataset, with 4111/6136 (67%) categorized as low intensity (<15 sessions annually), 2024/6136 (33%) as medium or higher intensity of care (>15 per year). We use the PRAUC metric to compare the predictive performance of machine learning models trained on real CAMHS data versus those trained on synthetic CAMHS data across different input sizes. Given the positive class prevalence of 33%, the random chance baseline (PRAUC baseline) is 0.33. As Table 4 illustrates, increasing sample size consistently improved predictive performance for both real and synthetic data models. In the most data-scarce setting (619 records), the random forest model’s ability to classify medium- or higher-intensity care patients was limited (real data: average PRAUC=0.39; synthetic data: PRAUC=0.32), However, when we increase the training set size to 6184 records, the model performance improved, with the average PRAUC for real data rising to 0.43 (a 30% increase over baseline) and for synthetic data to 0.40 (a 21% increase over baseline) demonstrating that the synthetic data preserves the analytical utility necessary to exceed the random threshold and capture real-world signals.

Our findings suggest that while privacy safeguards around children’s data are paramount and often lead to smaller datasets, the value of more data in building accurate and robust predictive tools cannot be overstated. Simply put, the more comprehensive the data, the better equipped the models are to provide meaningful insights that can ultimately support patient care.

**Table 4:** Comparison of random forest–based prediction models trained on real, tested on real (TRTR) Child and Adolescent Mental Health Services (CAMHS) data alone versus those trained on synthetic, tested on real (TSTR) CAMHS data.

Sample size	TRTR, PRAUC^a^ (range)	TSTR, PRAUC (range)
619 referrals	0.39 (0.34-0.44)	0.32 (0.28-0.38)
1546 referrals	0.40 (0.36-0.47)	0.33 (0.29-0.38)
3092 referrals	0.41 (0.37-0.47)	0.34 (0.30-0.39)
6184 referrals	0.43 (0.38-0.49)	0.40 (0.35-0.45)

^a^PRAUC: precision-recall area under the curve.

### Reproducibility of Feature Interpretability (SHAP)

**Figure 4:**
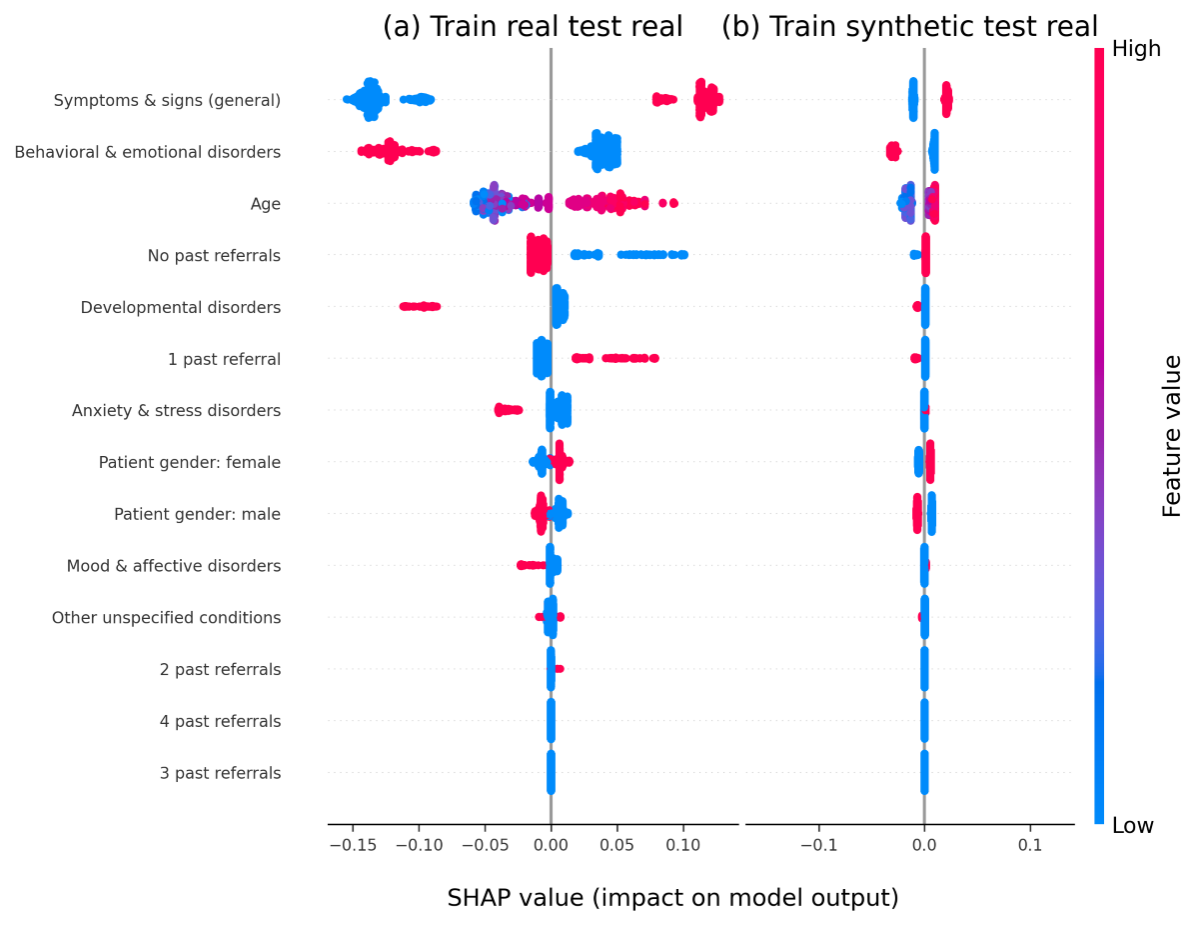
Shapley additive explanations (SHAP) values for intensity of care in (A) train real test real and (B) train synthetic test real models (n=6184).

SHAP analysis, presented in Figure 4, provides an insight into how different patient characteristics impact the predicted intensity of care, both when the model is trained on real patient data and when it is trained on synthetic patient data.

The model consistently showed that a lower prevalence of “behavioral and emotional disorders” (F90-F98) led to a higher predicted intensity of care. This finding is a clinically meaningful observation rooted in the structure of CAMHS resource allocation; because F90-F98 diagnoses (eg, ADHD, externalizing difficulties) are highly prevalent and typically managed via lower-intensity, first-line support, their presence is not a strong predictor of high-intensity service use. Therefore, the absence of this common, low-intensity diagnosis in a patient referred for complex care suggests the presence of a less common, more severe, or highly comorbid condition that immediately necessitates the allocation of higher-intensity resources [27].

Conversely, a greater presence of “symptoms and signs (general)” (R40-R46) or “developmental disorders” (F80-F89) contributed to a lower predicted intensity of care. Patient age also presented a clear and consistent trend; younger patients were generally predicted to require higher intensity care, while older patients were associated with lower predicted intensity. On the plots, lower values for a feature are typically shown in gray, higher values in black; features increasing predicted intensity extend to the right, while those decreasing it extend to the left.

While the models showed consistent variable impact on the target prediction (intensity of care), we noted a significant difference in the strength of this impact between the synthetic and the real prediction models. The model trained on actual patient data showed a much wider range of impacts from individual features (SHAP values ranging from approximately –0.15 to 0.15), suggesting that real patient characteristics could lead to more pronounced shifts in the predicted intensity of care. In contrast, the model trained on synthetic data displayed a significantly narrower range of influence (approximately –0.03 to 0.03). The “flattening” of the variables’ impact on the predicted intensity of care implies that while synthetic data helps the model learn the general direction of how patient characteristics relate to care intensity, it reduces the overall sensitivity of the model to the nuanced, individual variations found in real patient data. This effect, however, can be mitigated by using more advanced synthetic data generation techniques.

Despite the observed reduced variable impact in the synthetic model, the consistency in the direction of SHAP-based explanations remains clinically valuable. It effectively validates synthetic data’s ability to capture fundamental predictive patterns in sensitive health care records, making synthetic datasets particularly useful for privacy-preserving exploratory research and facilitating hypothesis generation and validation. While acknowledging the continued need for further efforts to precisely replicate nuanced quantitative impacts for high-stakes clinical predictions, the qualitative fidelity of the result offers an effective tool for initial understanding and safe experimentation in CAMHS health research.

### Synthetic and Real Individual Care Paths

**Figure 5:**
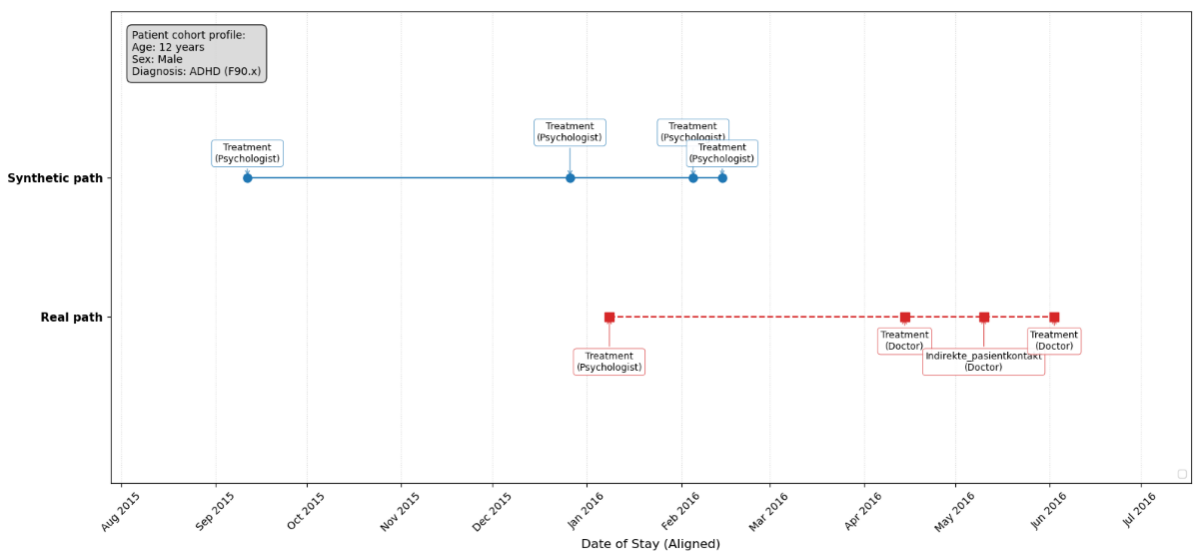
Comparison of real-like and synthetic care paths for an attention-deficit/hyperactivity disorder (ADHD) patient. The x-axis (Date of stay [Aligned]) shows the progression of time. The y-axis separates the "Synthetic path" (top, solid line) from the "Real path" (bottom, dashed line). Individual markers represent stay events, annotated with stay types and personnel categories. The grey box (top left) indicates shared patient demographics for both trajectories.

Figure 5 compares 2 distinct care pathways for a 12-year-old male patient diagnosed with “behavioral and emotional disorders” (F90-F98). Both paths are presented on a shared timeline (x-axis) that has been aligned to start at a common point, allowing for direct comparison of event sequencing and density.

The figure serves as a comparative visualization, highlighting potential differences in the flow, types of interventions, and personnel involvement between a synthetic and a more real care path.

While synthetic CAMHS data successfully captures the overarching statistical patterns of the real CAMHS dataset, divergences at the individual patient level offer valuable insights. The differences result from synthetic data representing the most common trajectories, an average derived from the entire dataset that naturally filters out the unique noise and unobserved idiosyncratic factors influencing real-world care. Real CAMHS care is inherently nonlinear and dynamic, shaped by patient responses, unforeseen crises, resource constraints, and patient/family agency. These complexities, including feedback loops and real-world bottlenecks such as waiting lists, are challenging for synthetic data to perfectly replicate at an individual level, leading to the observed divergence.

## Discussion

### Principal Findings

This study demonstrates the significant potential of synthetic data to enhance the utility of electronic health care records within CAMHS. Our methodology, using a hierarchical SDG, generated synthetic datasets that exhibited high statistical fidelity to real-world CAMHS data, as confirmed by various quality metrics (KSC/TVC, CS, CSS) and visual density comparisons. While minor quality variations were observed with decreasing sample sizes [28], the results confirm that our approach broadly preserves variable behaviors and dependencies between variables in the generated synthetic datasets [29]. However, identifying the precise minimum real data sample size for generating truly useful and high-fidelity synthetic CAMHS data is complex and highly dependent on the specific research scope and applications.

Models trained on synthetic data demonstrated predictive performance in classifying patient “intensity of care” (low vs medium or higher) that was comparable to models trained on real data. While the absolute predictive power of the model was modest (PRAUC=0.40-0.43 over a 0.33 random baseline), the key finding is the preservation of this utility in the synthetic data, as evidenced by the comparable scores**.**

The SHAP analysis confirmed that the synthetic model’s explainability aligned with real-world insights, underscoring the consistent importance of age and CAMHS diagnosis (eg, anxiety disorders, depression, ADHD, and autism spectrum disorder) in assessing care needs [30,31]. Furthermore, our findings provide strong evidence that synthetic data effectively protects individual privacy. The data generation process makes it highly unlikely for attackers to reidentify patients or uncover sensitive details from the original dataset, a critical requirement given the highly personal nature of CAMHS data.

The implications of high-fidelity, privacy-preserving synthetic CAMHS datasets are profound for children and adolescent mental health research and practice. Their availability removes critical barriers to data sharing and collaborative research, enabling more thorough investigations into mental health aspects across diverse populations [32], especially for vulnerable ones like those served by CAMHS. This capability facilitates a deeper understanding of mental disorder patterns and contributes to the development of more effective, personalized treatment methodologies [33]. Moreover, given that young adolescents often withhold important information from health care providers due to privacy concerns, which hinders effective diagnosis and treatment [34], synthetic data allows researchers to explore critical questions that would be impossible with real, incomplete data. These include understanding the impact of stigma on treatment adherence, evaluating novel personalized interventions, investigating the influence of “hidden” family dynamics, and simulating the effects of systemic barriers on access to care for vulnerable groups.

Furthermore, maintaining highly accurate long-term temporal and sequential relationships across numerous event types remains challenging in synthetic data generation. Most synthetic data quality metrics prioritize aggregate statistical properties over the exact fidelity of unique individual sequences. This means a synthetic dataset can be excellent at the population level but still show microlevel divergence in individual trajectories. Despite these differences, the divergence is valuable. It highlights that synthetic data, while not predicting exact individual pathways, excels at understanding general care models, developing system-level interventions via cohort simulations, and creating privacy-preserving case studies for training. It effectively complements the detailed study of highly personalized real-world clinical journeys.

### Future Work

To improve upon the findings of this investigation, future research can focus on enhancing the synthetic data generation process. Conditional generative models, such as variational autoencoders [35] and generative adversarial networks [36], could be trained to produce synthetic data that reflects specific referral reasons or treatment modalities, conditioned on existing basic patient information. These advanced models are adept at learning complex data distributions, leading to higher-quality and more diverse synthetic outputs, which are crucial for capturing the varied presentations within CAMHS.

Beyond static data generation, further research can also explore integrating synthetic data with process mining techniques to enhance the modeling of complex care sequences. Specifically, synthetic process mining offers a powerful avenue for generating and analyzing event logs that mimic real patient journeys, but with full privacy control [37]. This would enable the creation of highly detailed, longitudinal synthetic care pathways, allowing researchers to simulate and analyze “what if” scenarios for process optimization without touching real patient data (eg, assessing the impact of adding a new therapy step or reducing wait times on patient flow). Furthermore, synthetic process mining could also generate diverse and comprehensive training data for predictive models focused on patient trajectories and outcomes, especially for rare events or complex multistage interventions where real CAMHS data are scarce or incomplete. This capability would significantly advance our understanding of care delivery dynamics and improve operational efficiency in CAMHS, ultimately benefiting young people awaiting and receiving support.

### Limitations

It is important to recognize that our dataset had limitations in terms of clinical parameters. Our analysis revealed modest predictive power in the classification model (PRAUC approximately 0.43 on real data and 0.40 on synthetic data), necessitating caution in interpreting the absolute predictive accuracy. While the primary goal of this work was to establish the comparable utility between the synthetic and real datasets—which the similar PRAUC scores confirm—the absolute performance remains constrained. This constraint is likely attributable to 2 factors: severe class imbalance in the target variable (necessitating the aggregation of the rare high-intensity category into the 33% positive class) and the inherently limited, aggregated feature set available from administrative EMR. These limitations emphasize that the model should not be used in isolation for clinical prediction but rather that the synthetic data successfully preserves the utility inherent in the original feature space.

Furthermore, our dataset had limitations in terms of clinical parameters. Although we had access to basic data such as sex, age, and clinical primary diagnosis, our dataset lacked detailed information on the specific reason for the referral and the amount of additional care (eg, medication, group sessions, art therapy, physical therapy, and sheltered workshop). These clinical details are crucial for accurately assessing and classifying service intensity. The absence of these detailed clinical parameters may have impacted the accuracy of our analyses and the general applicability of our findings to a broader clinical context. Future research will benefit from a more comprehensive dataset that includes these key clinical parameters.

Regarding fairness evaluation, Norway’s highly inclusive public health care system inherently minimizes selection bias. However, we acknowledge the potential for underrepresentation of underserved minorities and rare diagnoses. These issues can be effectively addressed by augmenting the data with synthetic samples of these specific groups and applying fairness measures tailored to the analytical goals of research aimed at serving these minority populations. While the methodology for creating synthetic CAMHS data is highly generalizable across different data environments and health care systems, it is crucial to recognize that the interpretation and applicability of findings derived from such synthetic data would always need to be contextualized within the specific health care system from which the original data was sourced. This underscores the need for localized validation and careful consideration of systemic differences when applying research findings across international borders.

### Conclusions

This study provides evidence that synthetic data can be generated from CAMHS EMR that achieves a favorable balance between utility and privacy. The main findings confirm that the hierarchical synthetic data model produces datasets with high statistical fidelity and comparable predictive utility to real data, while simultaneously demonstrating a reduced risk of patient reidentification. This pilot study suggests that synthetic data, developed from standard EMR clinic records, offers a method compliant with HIPAA and GDPR regulations, facilitating clinical and administrative research that was previously unattainable. This advancement creates critical new opportunities for CAMHS research to progress despite confidentiality restrictions.

## Appendix

Multimedia Appendix 1 Entity-relationship diagram of the patient data schema. This diagram illustrates the relationship between the "referral" and "stay" entities, detailing the attributes and their data types (ID, datetime, numerical, categorical) for each, along with their respective primary and foreign keys.

Multimedia Appendix 2 Synthetic generation process. We used a constant input for the synthetic data generator HMSynthetiser (ie, a fixed subsample from the real dataset) to output 4 different synthetic CAMHS data. The similarity between each of the datasets was compared to the real training data by performing KSC/TVC, CS, and CSS metrics via the SDMetrics Python package.

Multimedia Appendix 3 Privacy evaluation. The Main attack (1) uses synthetic data to create a series of guesses. Anonymeter evaluates (2) these guesses by comparing them to the values present in the original dataset, to assess the effectiveness of the attack. Anonymeter requires the adversary to conduct the same attack on a control dataset (control attack), which consists of records that were not utilized in the generation of the synthetic data. Any insights the attacker gains regarding the control records from the synthetic dataset cannot be linked to privacy violations, as the design ensures that no information from the control dataset is incorporated into the synthetic dataset. By contrasting the success rates of the attack on both training records and control records, a sensitive measure of risk (3) is calculated.

Multimedia Appendix 4 Train synthetic test real. To evaluate model efficacy, the train synthetic test real (TSTR) approach was used. Two machine learning models were developed: one trained on real data and the other on synthetic data, both targeting patients with referral duration under 18 months to predict Child and Adolescent Mental Health Services (CAMHS) care intensity across three categories: fewer than 15 sessions, 15 or more sessions annually. The latter two categories were combined due to low occurrence of high intensity. Model performance was assessed using a 20% test subset from the real dataset, allowing comparison of synthetic and real data effectiveness. SHAP values were computed to analyze feature significance.

Multimedia Appendix 5 Characteristics of the training (n=6132) and test (n=1531) datasets. This table presents the distribution of demographic, clinical, and diagnostic variables across the training and test cohorts.

## Abbreviations

ADHD: attention-deficit/hyperactivity disorder
CAMHD: Child and Adolescent Mental Health Department
CAMHS: Child and Adolescent Mental Health Services
CS: CorrelationSimilarity
CSS: CardinalityShapeSimilarity
DIPS: Distrubuert Informasjons og Pasientdatasystem i Sykehuset
EMR: electronic medical record
GDPR: General Data Protection Regulation
HIPAA: Health Insurance Portability and Accountability Act
ICD-10: International Statistical Classification of Diseases, Tenth Revision
KSC: Kolmogorov-Smirnov Complement
PRAUC: precision-recall area under the curve
SDG: synthetic data generator
SHAP: Shapley additive explanations
SUS: Stavanger University Hospital
TVC: Total Variation Complement

## Data Availability

The underlying dataset can be shared upon reasonable request after signing a data transfer agreement with the receiving institution to ensure the protection of personal information. The code underlying these analyses is available from GitHub [38].

## References

[1] Aboujaoude E (2019). Protecting privacy to protect mental health: the new ethical imperative. J Med Ethics.

[2] Iflaifel M, Hall CL, Green HR, Willis A, Rennick-Egglestone S, Juszczak E, Townsend M, Martin J, Sprange K (2023). Widening participation - recruitment methods in mental health randomised controlled trials: a qualitative study. BMC Med Res Methodol.

[3] Berardi C, Antonini M, Jordan Z, Wechtler H, Paolucci F, Hinwood M (2024). Barriers and facilitators to the implementation of digital technologies in mental health systems: a qualitative systematic review to inform a policy framework. BMC Health Serv Res.

[4] Henderson C, Noblett J, Parke H, Clement S, Caffrey A, Gale-Grant O, Schulze B, Druss B, Thornicroft G (2014). Mental health-related stigma in health care and mental health-care settings. Lancet Psychiatry.

[5] Sedlakova J, Daniore P, Horn Wintsch A, Wolf M, Stanikic M, Haag C, Sieber C, Schneider G, Staub K, Alois Ettlin D, Grübner O, Rinaldi F, von Wyl V, University of Zurich Digital Society Initiative (UZH-DSI) Health Community (2023). Challenges and best practices for digital unstructured data enrichment in health research: a systematic narrative review. PLoS Digit Health.

[6] Waithira N, Mukaka M, Kestelyn E, Chotthanawathit K, Thi Phuong DN, Thanh HN, Osterrieder A, Lang T, Cheah PY (2024). Data sharing and reuse in clinical research: are we there yet? A cross-sectional study on progress, challenges and opportunities in LMICs. PLOS Glob Public Health.

[7] National Academies of Sciences, Engineering, and Medicine (2013). Frontiers in Massive Data Analysis.

[8] Bhanot K, Qi M, Erickson JS, Guyon I, Bennett KP (2021). The problem of fairness in synthetic healthcare data. Entropy.

[9] Begoli E, Brown K, Srinivas S, Tamang S (2022). SynthNotes: a generator framework for high-volume, high-fidelity synthetic mental health notes.

[10] Mori S, Ignat O, Lee A, Mihalcea R (2024). Towards algorithmic fidelity: mental health representation across demographics in synthetic vs. human-generated data. arXiv. Preprint posted online on March 25, 2024.

[11] van Breugel B, Kyono T, Berrevoets J, van der Schaar M (2021). Decaf: generating fair synthetic data using causally-aware generative networks.

[12] Kamiran F, Calders T (2022). Classifying without discriminating.

[13] Ramachandranpillai R, Sikder MF, Bergström D, Heintz F (2024). Bt-GAN: generating fair synthetic healthdata via bias-transforming generative adversarial networks. J Artif Intell Res.

[14] Rankin T, Gonzales M (2022). Evaluating predictive models trained on synthetic data for mental health applications. Artif Intell Med.

[15] Ajith M, Aycock DM, Tone EB (2024). A deep learning approach for mental health quality prediction using functional network connectivity and assessment data. Brain Imaging Behav.

[16] Lee K, Kim H (2023). Machine learning for passive mental health symptom prediction: generalization across different longitudinal mobile sensing studies. PLoS One.

[17] Patki N, Wedge R, Veeramachaneni K (2022). The synthetic data vault.

[18] Houssou R, Augustin M, Rappos E, Bonvin V, Robert-Nicoud S (2022). Generation and simulation of synthetic datasets with copulas. arXiv. Preprint posted online on March 30, 2022.

[19] Giomi M, Boenisch F, Wehmeyer C, Tasnádi B (2022). A unified framework for quantifying privacy risk in synthetic data. arXiv. Preprint posted online on November 18, 2022.

[20] Lu Y, Chen L, Zhang Y, Shen M, Wang H, Wang X (2023). Machine Learning for Synthetic Data Generation: A Review. arXiv. Preprint posted online on February 8, 2023.

[21] Beckers T, Koekkoek B, Tiemens B, Hutschemaekers G (2023). Measuring the intensity of mental healthcare: Development of the Mental Healthcare Intensity Scale (MHIS). BMC Psychiatry.

[22] Uddin S, Khan A, Hossain ME, Moni MA (2019). Comparing different supervised machine learning algorithms for disease prediction. BMC Med Inform Decis Mak.

[23] Esteban C, Hyland S, Rätsch G (2017). Real-Valued (MEDICAL) time series generation with recurrent conditional GANs. arXiv. Preprint posted online on June 8, 2017.

[24] Kiran A, Kumar S, So-In C, Londhe ND, Bhatt N, Kitsing M (2023). Synthetic data and its evaluation metrics for machine learning. Information Systems for Intelligent Systems. Smart Innovation, Systems and Technologies.

[25] Richardson E, Trevizani R, Greenbaum JA, Carter H, Nielsen M, Peters B (2024). The receiver operating characteristic curve accurately assesses imbalanced datasets. Patterns (NY).

[26] Lundberg S, Lee S (2017). A unified approach to interpreting model predictions. https://proceedings.neurips.cc/paper_files/paper/2017/file/8a20a8621978632d76c43dfd28b67767-Paper.pdf.

[27] Edbrooke-Childs J, Rashid A, Ritchie B, Deighton J (2023). Predictors of amounts of child and adolescent mental health service use. Eur Child Adolesc Psychiatry.

[28] McDermott MBA, Wang S, Marin N (2020). A closer look at AUROC and AUPRC under class imbalance.

[29] Marin J (2022). Evaluating synthetic tabular data generated to augment small sample datasets. arXiv. Preprint posted online on November 19, 2022.

[30] Benali F, Bodénès D, Labroche N, de RC (2021). MTCopula synthetic complex data generation using copula. https://ceur-ws.org/Vol-2840/paper8.pdf.

[31] Smith J, Doe A (2023). Age and Developmental Considerations in CAMHS. J Child Psychol Psychiatry.

[32] Brown L, Green P (2024). Gender differences in mental health diagnoses and treatment. J Adolesc Health.

[33] Rocks S, Stepney M, Glogowska M, Fazel M, Tsiachristas A (2018). Understanding and evaluating new models of Child and Adolescent Mental Health Services in South-East England: a study protocol for an observational mixed-methods study. BMJ Open.

[34] (2018). Improvements ahead: how humansAI might evolve together in the next decade. Pew Research Center.

[35] Wan Z, Zhang Y, He H (2017). Variational autoencoder based synthetic data generation for imbalanced learning.

[36] Guo J, Jin S, Wang M, Han C (2023). Generating synthetic mixed-type longitudinal electronic health records for artificial intelligent applications. NPJ Digit Med.

[37] Bullward A, Aljebreen A, Coles A (2022). Process mining and synthetic health data: reflections and lessons learnt.

[38] mounirHai/SynthCAMH. GitHub.

