# Supplementary figures and images for: Balancing Privacy and Utility in Child and Adolescent Mental Health Services Research: Retrospective Cohort Study on Synthetic Data Generation
Source: JMIR Med Inform. 2026 Feb 26;14:e71819. doi: 10.2196/71819 (PMC12982954; doi:10.2196/71819)

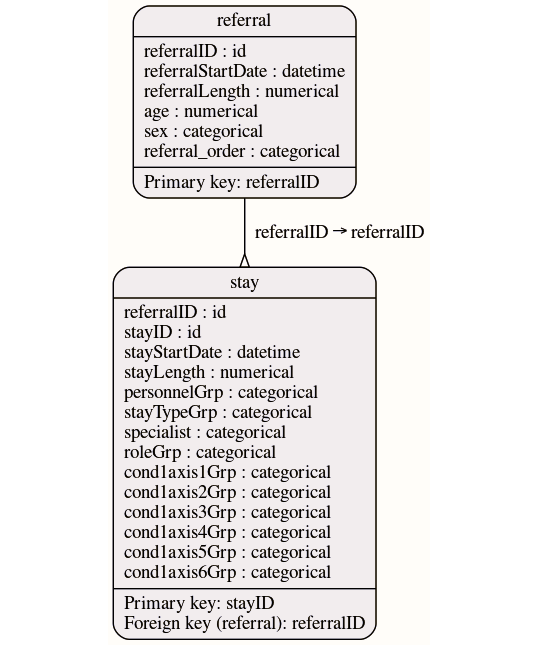

Supplement: Multimedia Appendix 1 [file medinform_v14i1e71819_app1.png]

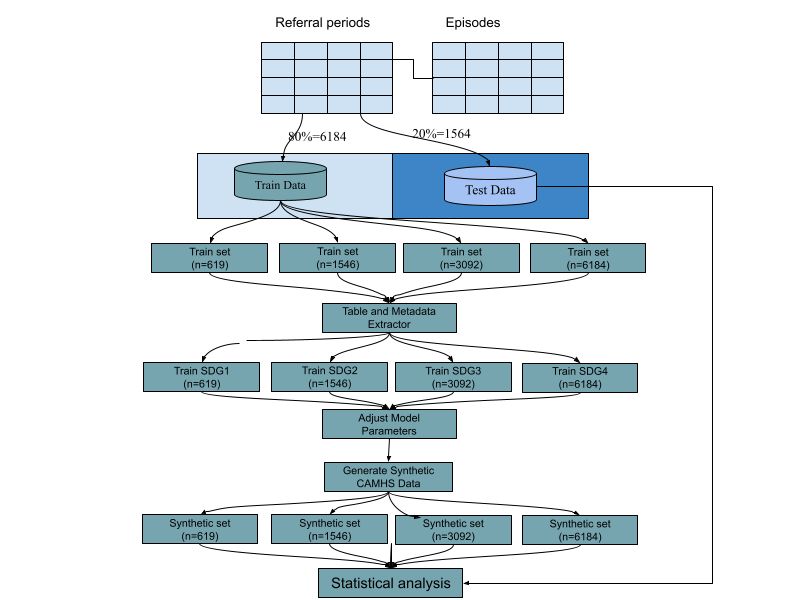

Supplement: Multimedia Appendix 2 [file medinform_v14i1e71819_app2.png]

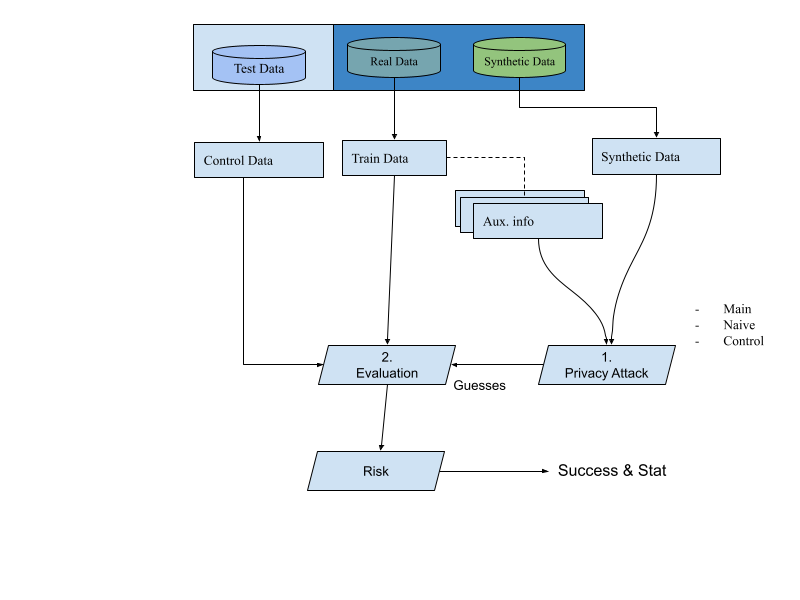

Supplement: Multimedia Appendix 3 [file medinform_v14i1e71819_app3.png]

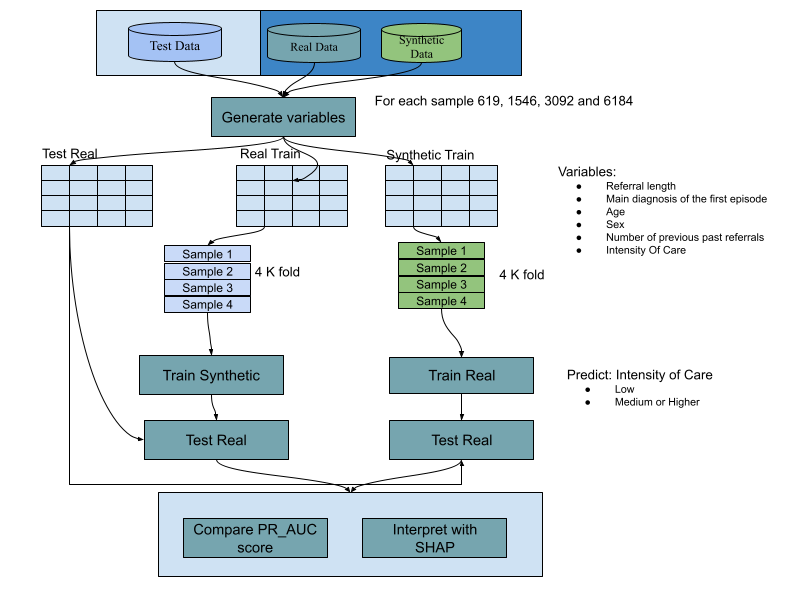

Supplement: Multimedia Appendix 4 [file medinform_v14i1e71819_app4.png]
