# Supplementary material for: Balancing Privacy and Utility in Child and Adolescent Mental Health Services Research: Retrospective Cohort Study on Synthetic Data Generation
Source: JMIR Med Inform. 2026 Feb 26;14:e71819. doi: 10.2196/71819 (PMC12982954; doi:10.2196/71819)
Supplement: Multimedia Appendix 5 [file medinform_v14i1e71819_app5.pdf]

Characteristic	Training (N=6184)	Test (N=1564)
**Demographic Variables**		
Age		
Mean age in years	11.72	11.58
Range, years	1-20	1-20
Gender, n (%)		
Male	3168 (51.7)	823 (53.8)
Female	2968 (48.3)	708 (46.2)
**Clinical Variables**		
Intensity of Care, n (%)		
Low	4138 (67.5)	1033 (67.5)
Medium	1674 (27.3)	418 (27.3)
High	324 (5.3)	80 (5.2)
Number of past referrals, n (%)		
0	5504 (89.7)	1362 (89.0)
1	566 (9.2)	162 (10.6)
≥2	66 (1.1)	7 (0.5)
Number of episodes per referral		
Median (IQR)	6 (3-13)	6 (2-12)
Referral length in days		
Median (IQR)	362 (165-678)	363 (167-651)
**Diagnostic Variables**		
Primary Diagnosis of the first episode, n (%)		
Cognitive, Emotional & Behavioral Symptoms	1740 (28.4)	421 (27.5)
Health Service Encounters (Exam/Invest.)	1411 (23.0)	362 (23.6)
Child/Adolescent Behavioral & Emotional Disorders	1147 (18.7)	302 (19.7)
Neurotic, Stress & Somatoform Disorders	569 (9.3)	125 (8.2)
Mood Disorders	258 (4.2)	63 (4.1)
Psychological Developmental Disorders	313 (5.1)	81 (5.3)
Other	1269 (20.7)	177 (11.6)
